# Enhancing Surface Hygiene in Healthcare with GNP@PEGDA Composites for Antibacterial and Photothermal Applications

**DOI:** 10.1021/acsomega.5c09656

**Published:** 2025-11-11

**Authors:** Shih-Mao Peng, Lutvi Vitria Kadarwati, Yueh-Ying Hsieh, Muhammad Saukani, Jen-Chang Yang, Tsung-Rong Kuo

**Affiliations:** † Graduate Institute of Biomedical Materials & Tissue Engineering, 38032 Taipei Medical University, Taipei City 110, Taiwan; ‡ International Ph.D. Program in Biomedical Engineering, College of Biomedical Engineering, 38032 Taipei Medical University, Taipei City 110, Taiwan; § Department of Orthopaedics, Shuang Ho Hospital, 38032 Taipei Medical University, New Taipei City 235, Taiwan; ∥ Department of Orthopaedics, School of Medicine, College of Medicine, 38032 Taipei Medical University, Taipei City 110, Taiwan; ⊥ Department of Orthopedics, Hsin Kuo Min Hospital, 38032 Taipei Medical University, Taoyuan City 320, Taiwan; # Department of Mechanical Engineering, Faculty of Engineering, Universitas Islam Kalimantan MAB, Banjarmasin 70124, Kalimantan Selatan, Indonesia; ∇ Graduate Institute of Nanomedicine and Medical Engineering, College of Biomedical Engineering, 38032 Taipei Medical University, Taipei City 110, Taiwan

## Abstract

The state of hygiene of healthcare materials and facilities is critical for the safety of both patients and healthcare workers. Addressing the emergence of drug-resistant bacteria underscores the need to develop new antibacterial treatment methods. Photothermal technology offers a novel and promising approach by utilizing visible light or ultraviolet irradiation for healthcare facilities and equipment, whereby thermal effects or photothermal reactions are generated to eliminate surface bacteria. This method not only achieves efficient sterilization but also reduces reliance on chemical disinfectants, thereby minimizing environmental burdens. Poly­(ethylene glycol) diacrylate (PEGDA), a polymer commonly used in stereolithography (SLA) 3D printing, lacks the ability to absorb infrared radiation and induce photothermal reactions. To overcome this limitation, graphene nanoplates (GNPs), which exhibit excellent infrared absorption properties, were blended with PEGDA to create a composite material capable of infrared radiation absorption. Using SLA, a 3D printing technology, porous structures were fabricated to enhance the heat absorption efficiency, thus reducing material consumption. The resulting material, GNP@PEGDA, demonstrated promising photothermal performance and improved sterilization efficiency. This research highlights the potential of combining surface antibacterial and photothermal technologies to improve the hygiene of healthcare materials and facilities. The findings provide a promising pathway for developing innovative solutions to combat drug-resistant bacteria and enhance sterilization methods.

## Graphical abstract

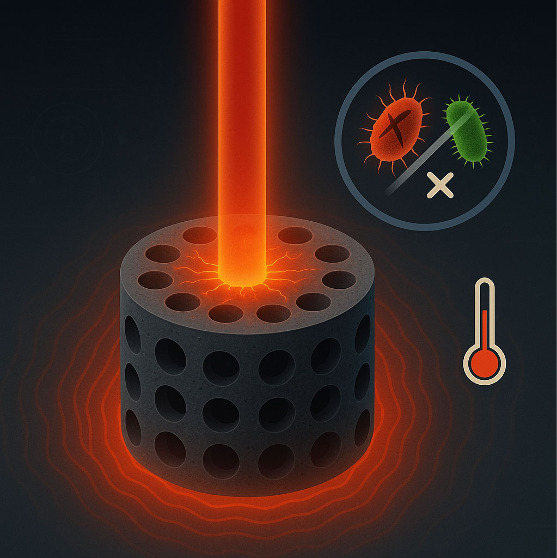

## 1 Introduction

There are environment needs to be aseptic, encompassing surgeries, medical equipment, and healthcare settings like hospitals and nursing centers. However, the challenges of infections in aseptic environments, aided by highly adaptable and rapidly proliferating bacteria such as *Mycobacterium tuberculosis* (Mtb),^1,2^ *Staphylococcus aureus* (*S. aureus*),^3–5^ and *Escherichia coli* (*E. coli*),^6–8^ persistently pose significant problems.^9–12^ Currently, several strategies have been developed to combat bacterial infections, including methods such as releasing reactive oxygen species (ROS);^13–18^ synthesizing a silver–palladium bimetallic alloy nanocage, AgPd0.38, with enzymatic properties similar to oxidases; utilizing surface ROS to effectively eliminate antibiotic-resistant bacteria, thereby delaying the onset of bacterial resistance; using photothermal therapy (PTT);^19–24^ synthesizing a water hydrogel HT/GGA/GO with a porous cross-linked network structure, which served as a functional dressing with an injectable hyaluronic acid hydrogel, that possessed antioxidant and photothermal antibacterial activities;^25^ releasing metal ions from nanoparticles (NPs); and preparing a zinc-based metal–organic framework (MOF) with benzoic hydrazide linkers as an antibacterial material against the Gram-positive bacterium, *S. aureus*. This material inhibits bacterial growth and metabolic activity with an approximate half-maximal effective concentration of 20 mg/L when dispersed in culture medium.^26^

The basic principle of three-dimensional (3D) printing is to create 3D objects on a digital model. 3D printing belongs to additive manufacturing, which reduces material waste, lowers production costs for prototypes and small batches, and achieves rapid product development cycles. 3D printing is extensively being used in lots of applications, from the bioprinting of organ tissues to the industrial printing of houses. As 3D printing technology continues to evolve, it has the potential to reshape industrial processes to open new realms of creativity.^27–29^

The photothermal antibacterial effect has significantly advanced in recent years, offering a promising option for achieving sterilization in medical environments, wound disinfection, and even tumor eradication.^30–32^ The previous literature reports various photothermal biomaterials,^33–35^ including metal NPs,^36–38^ MOFs,^39–41^ carbon nanomaterials,^42–44^ transition metal sulfides,^45–47^ and near-infrared (NIR)-absorbing complex materials.^48–51^ A porous scaffold was fabricated with poly-l-lactide acid (PLLA)/zeolitic imidazolate framework-8@graphene oxide (ZIF-8@GO) using 3D printing. This scaffold can facilitate photothermal effects and release zinc ions from ZIF-8 to kill bacteria, while PLLA aids in tissue repair.^52^ Forsterite scaffolds were successfully fabricated by combining 3D printing with a polymer-derived ceramic (PDC) strategy.^53,54^ Through this approach, free carbon is formed in the forsterite scaffolds, enhancing the photothermal performance. This property provides forsterite scaffolds with photothermal-induced antibacterial activity, making them effective in preventing bacterial infections in bone defects.

In this study, we introduce production of 3D printing ink using a mixture of poly­(ethylene glycol) diacrylate (PEGDA) and water, into which a small amount of carbon powder, consisting of graphene nanoplatelets (GNPs), was incorporated to create a composite material.^55–58^ While the PEGDA-only system had no photothermal effect, we added 0.2 g GNPs to achieve rapid sterilization. Furthermore, by using 3D printing to create a porous structure, we further enhanced the heating rate, providing a new and feasible approach to achieve antibacterial effects in medical environments and the sterilization of equipment.

## 2 Experimental Section

### 2.1 Materials

PEGDA with an average *M*_n_ 700 was obtained from Sigma-Aldrich. Diphenyl­(2,4,6-trimethylbenzoyl)­phosphine oxide at 97% was obtained from Sigma. GNPs were obtained from Sigma-Aldrich.

### 2.2 Fabrication of 3D Printing Ink GNP@PEGDA

First, 0.2 g of diphenyl­(2,4,6-trimethylbenzoyl)­phosphine oxide at 97% was added to 75 mL of PEGDA (*M*_n_ 700), and the mixture was sonicated with ultrasound until the diphenyl was completely dissolved. Then, 25 mL of deionized water (DIW) and 0.2 g of GNPs were added, and the mixed solution was stirred at 1150 rpm at a temperature at 65 °C for 2 h to ensure an even distribution of GNPs and to prevent agglomeration.

### 2.3 Fabrication of 3D Printed GNP@PEGDA Scaffolds

The 3D model was created using SolidWorks. The 3D printing technology utilizes stereolithography (SLA) with a Formlabs 3+ 3D printer. The printer features a light processing unit (LPU) with a laser spot size of 85 μm, a laser wavelength of 405 nm, and a power of 250 mW. Each layer was set to a thickness of 100 nm. The building chamber temperature was heated to 35 °C. For solid (S) structure, the dimensions of the GNP@PEGDA cuboid (Cub) were 5 × 5 × 10 mm^3^ (GNP@PEGDA (Cub_S)), while the cylindrical (Cyl) dimension was a diameter of 5 mm and a height of 10 mm (GNP@PEGDA (Cyl_S)). For porous (P) structure, the GNP@PEGDA cuboid dimensions were 5 × 5 × 10 mm^3^ (GNP@PEGDA (Cub_P)), and the GNP@PEGDA cylindrical dimension was a diameter of 5 mm and a height of 10 mm (GNP@PEGDA (Cyl_P)).

### 2.4 Microbial Cultures

The microorganisms examined in this research were *E. coli* (hp α) and *S. aureus*. A 20 μL aliquot of each bacterium was cultured in 3 mL of trypticase soy broth (TSB) as the culture medium and incubated overnight in a shaker incubator under conditions of 37 °C and 170 rpm.

### 2.5 Antibacterial Activity Assay

*E. coli* and *S. aureus* were grown overnight. After 18–24 h, the optical density at 600 nm (OD_600_) of the bacterial solutions was observed and set to 0.2 by diluting the bacterial solution into 1.5 mL of new culture medium to determine the antibacterial effect. Different shapes of GNP@PEGDA were independently examined by adding the shapes into 1.5 mL of the latest bacterial solution and directly irradiating them together under NIR laser light for 10 min. Temperature changes were observed at this stage. Thereafter, the bacterial solutions were reincubated at 37 °C and 170 rpm. OD_600_ values were measured every 30 min for 3 h.

### 2.6 Preparation of Fixing Bacteria for Scanning Electron Microscopic (SEM) Observations

Fixing bacteria on silver glass was used to observe the bacterial morphology under SEM. Initially, 20 μL of *E. coli* or *S. aureus* was cultured with 3 mL of a TSB medium solution and incubated overnight. The next day, new medium of bacteria was set up by taking 1.5 mL of the TSB solution and adding bacteria until an optical density of 0.2 was achieved. For this treatment, all shapes of the GNP@PEGDA scaffolds were put into a new bacterial solution, irradiated under NIR light for 10 min, and incubated for 3 h. After culturing for 3 h, the bacterial solution was placed into an Eppendorf tube and centrifuged for 3 min at a speed of 3500 rpm. The upper layer of the solution was removed, and the bacterial deposit was retained in the tube. After that, 1 mL of DDI H_2_O was added to rinse the bacteria, shaken for a while until the bacteria were mixed, and centrifuged three times at the same duration and speed. Subsequently, 1 mL of 4% formaldehyde was added, shaken, and left for 1 h. Then, the bacteria were rinsed with a similar step as before but at a speed of 2500 rpm three times. In the subsequent stage, 1 mL of 70% ethanol was added to dilute the bacterial solution. For SEM observations, the bacteria that had been fixed were then dropped onto glass silicon and dried. The sample was ready to observe under an SEM instrument.

### 2.7 Agar Plate Assay

An agar plate was independently prepared by making a solution of LB medium, agar, and deionized water before being sterilized at 121 °C for 1 h. The solution was molded into agar plates and cooled for use in a temperature room. After that, 1.5 mL of a bacterial solution (OD = 0.2) was added to each shape of GNP@PEGDA and irradiated under NIR light for 10 min. At a ratio of 1:9, 167 μL of the bacterial solution was diluted into a new TSB medium with as much as 10^–1^ dilution for *E. coli* and 10^–2^ for *S. aureus*. A 20 μL aliquot of the mixture was dripped onto each agar plate and spread using glass beads. This step was undertaken with three replicates for each shape. All treated agar plates were incubated overnight at 37 °C. Observing and counting of the bacterial survival tests of GNP@PEGDA were done by employing ImageJ software.

## 3 Results and Discussion

### 3.1 Microstructure of 1 wt % GNP@PEGDA Scaffolds

By SEM, the microstructural characteristics of GNP powder and GNP@PEGDA were carefully examined and elucidated. In Figure 1a, the inherent structure of the original GNP powder was observed as an extremely thin, intricately convoluted, and evenly distributed flake-like structure, with each GNP flake having a diameter of approximately 10 μm, as depicted in Figure 1b. In stark contrast, the synthesized GNP@PEGDA sample, prepared by mixing GNP powder into a solution containing PEGDA resin specifically tailored for 3D printing ink, revealed multiple pores on the surface prepared from the 3D printing ink. The reason for this phenomenon could have been the powder obstructing the laser in the printing layers, resulting in some areas where the material was not printed (Figure 1c). However, upon magnifying the surface layer for observation, the GNP powder was seen to be uniformly dispersed inside, indicating that while the success of the material’s preparation might have been hindered to some extent, it did not affect the material’s distribution. Additionally, in Figure 1d, the powder was transformed into spherical granules. The cause of this transformation might be attributed to the smoothing process conducted after each layer’s printing in 3D printing, potentially disrupting the flake-like structure of the GNP powder, leading to its granular form. Notably, the ultraviolet–visible (UV–vis) spectrum in Figure 1e ultimately reveals that the PEGDA resin solution exhibited no discernible IR light absorption characteristics. The absorption peaks between 200 and 400 nm were attributed to the addition of a photosensitizer, leading to the appearance of these signals. The photosensitizer functions by absorbing UV light, thereby initiating the polymerization of high-molecular-weight resin, transforming it from a liquid state to a solid state. Consequently, PEGDA alone exhibited no absorption signals. However, introducing GNP powder into the solution accelerated the emergence of identifiable IR light absorption characteristics. Energy beyond 800 nm was absorbed by GNP, thus generating a photothermal effect.

**Figure 1:**
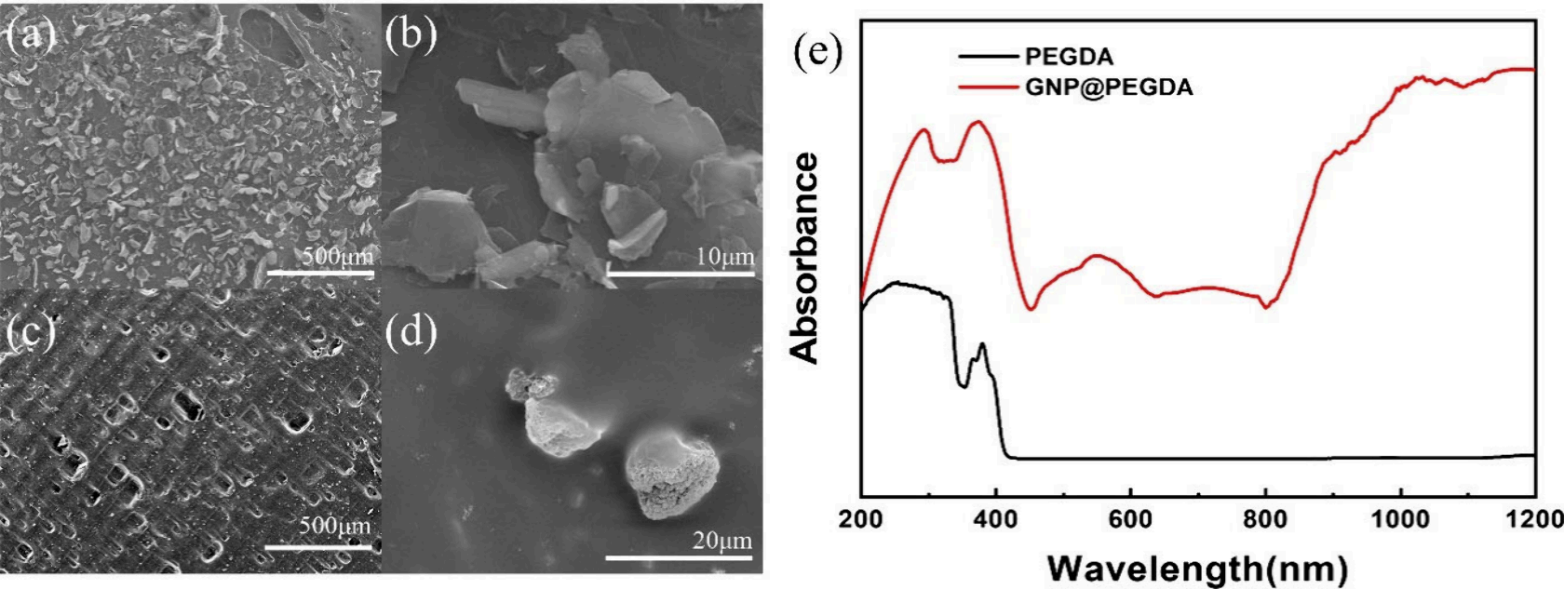
SEM images of GNP powder at (a) low and (b) high magnification. SEM images of GNP@ PEGDA at (c) low and (d) high magnification. (e) UV–vis absorption spectra of PEGDA and GNP@PEGDA.

### 3.2 Photothermal Performance of the Scaffolds

A detailed assessment of the photothermal effects on the scaffolds was carried out, depicting their susceptibility to NIR radiation, as carefully illustrated and intuitively depicted in Figure 2. When these scaffolds were judiciously exposed to the penetrating light beam of an NIR laser, a particularly intriguing and noteworthy phenomenon emerged. Specifically, under the influence of the NIR light source, the unaltered and original form of PEGDA exhibited no significant or pronounced temperature elevation over time, consistently remaining at 25 °C. However, in stark contrast, samples containing added GNP powder displayed a continuous and gradual temperature increase with prolonged and uninterrupted exposure to radiation emitted by the NIR light source. It was observed that the temperature of the solid material rose from 25 to 45 °C within the initial 4 min, demonstrating a rapid initial increase in temperature followed by stabilization. This remarkable phenomenon convincingly and distinctly demonstrated the extraordinary and substantial capability of GNP to efficiently convert incident NIR light energy into a potent and identifiable reservoir of heat energy. When transitioning the solid material into a porous structure, it was observed that under the same time frame, the porous structure exhibited a faster rate of temperature increase compared to the solid material. Whether in the form of cylindrical or cuboid porous structures, within the first 4 min, temperatures of around 55 °C were reached, which was approximately 10 °C higher than the solid material. These observed results significantly underscored and emphasized the superior rate of temperature increase and higher photothermal performance exhibited by the porous scaffold structures. This is because they cleverly harness and utilize the energy generated by NIR radiation to achieve widespread practical and real-world applications. Moreover, the porous structure provided a larger surface area compared to solid materials, facilitating enhanced sterilization options while reducing energy demands.

**Figure 2:**
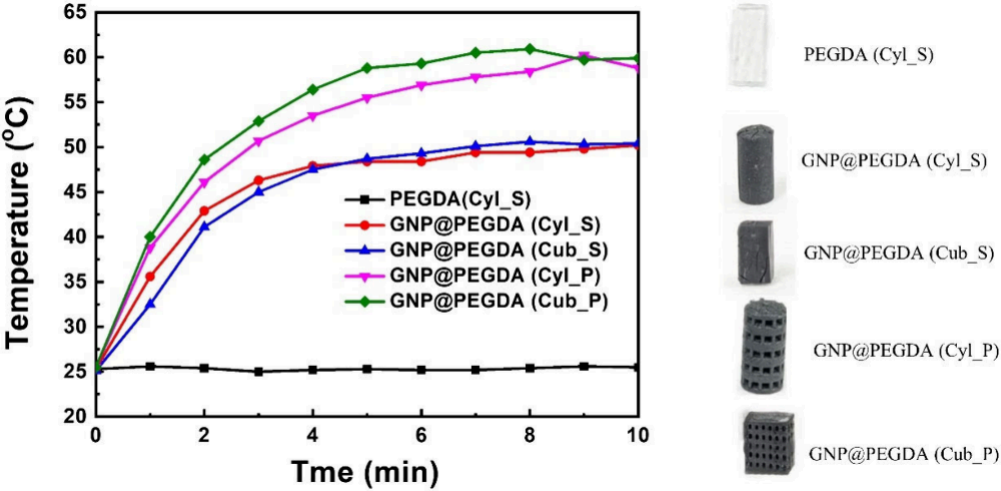
Temperature elevation curves of different shapes of the GNP@PEGDA and PEGDA sample scaffolds. PEGDA (Cyl_S): PEGDA with a cylindrical and solid structure. GNP@PEGDA (Cyl_S): GNP@PEGDA with a cylindrical and solid structure. GNP@PEGDA (Cub_S): GNP@PEGDA with a cuboid and solid structure. GNP@PEGDA (Cyl_P): GNP@PEGDA with a cylindrical and porous structure. GNP@PEGDA (Cub_P): GNP@PEGDA with a cuboid and porous structure.

### 3.3 In Vivo Photothermal Activity after NIR Radiation

The photothermal effect was created by irradiating a sample for 10 min under an NIR laser at a power density of 2.0 W/cm^2^. To validate the photothermal efficiencies, different shapes of GNP@PEGDA were irradiated, including a solid cylinder, porous cylinder, solid cuboid, and porous cuboid. Figure 3a shows that the temperature of the control group slightly increased from 27 to 31 °C with 10 min NIR laser irradiation. In comparison, the temperature of *E. coli* cultured with scaffolds of different shapes began to increase in the first minute to 40 °C and steeply rose until it reached 63 °C, particularly for the solid cuboid. Moreover, a continuous increase in temperature was observed for the four scaffolds. This can be attributed to the slight evaporation of the bacterial solution during near-infrared laser irradiation. Additionally, the TSB group in Figure 3b remained unchanged at 30 °C after irradiation. Meanwhile, the temperature in the bacterial solution of *S. aureus* incubated with GNP@PEGDA scaffolds also evenly increased to 45 °C in the initial 60 s. The trend tended to increase in all four scaffold forms and reached peaks of 63–73 °C. The solid forms of the cylinder and cuboid reached the highest temperatures with *S. aureus* of 70 and 72 °C, respectively. This was a consequence of the solid form conducting heat better compared to the porous form.

**Figure 3:**
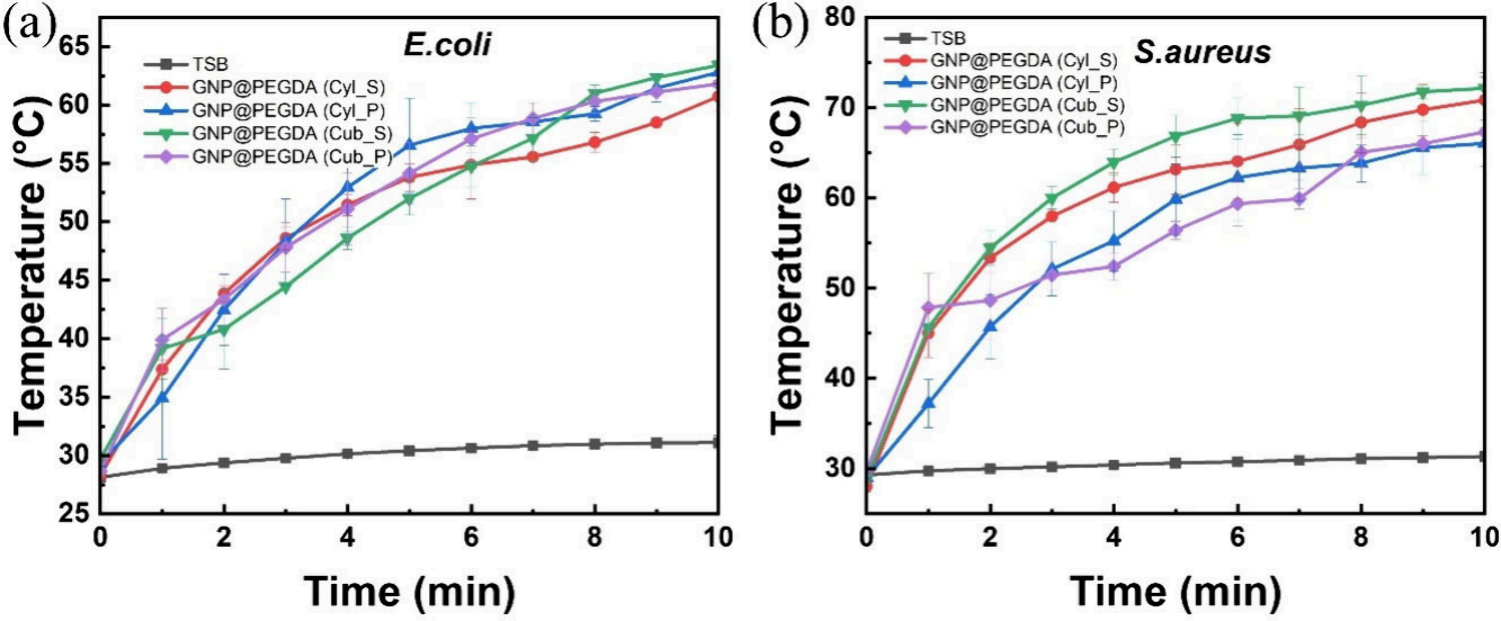
Photothermal activity after irradiation of (a) *Escherichia coli* (*E. coli*) and (b) *Staphylococcus aureus* (*S. aureus*).

### 3.4 Antibacterial Activity of GNP@PEGDA without NIR Radiation

Antibacterial activity is generally related to substances that kill bacteria or decrease their rate of growth without being hazardous to neighboring tissues. Figure 4 shows antibacterial activity without irradiation. It was found that there was no effect of GNP@PEGDA scaffolds unless they were accompanied by laser light absorption. In the OD growth curves of *E. coli* and *S. aureus* (Figure 4a,b), those bacteria began to grow in the first 30 min, then significantly increased from 30 to 180 min. After 3 h, the OD of *S. aureus* showed rapid growth to a maximum OD of 2.0 for almost all forms incubated with bacteria compared to *E. coli* which only rose to 1.4 in the porous cuboid form. Moreover, to understand the antibacterial effect of GNP@PEGDA scaffolds, changes in cell morphologies and membranes of *E. coli* and *S. aureus* due to the treatment with GNP@PEGDA scaffolds but without light irradiation were further evaluated. As shown in Figure 4c, both *E. coli* and *S. aureus* bacterial cells were viable in the absence of GNP@PEGDA scaffolds, with no evidence of membrane damage or cell death. No change in shape was depicted compared to the control group with only TSB as observed by SEM. This revealed that the appropriate shape and morphology of *E. coli* presented a rod shape, while a spherical shape was shown by *S. aureus.* As a result, different shapes of GNP@PEGDA scaffolds did not affect bacterial growth either in terms of antibacterial activity or cell morphology.

**Figure 4:**
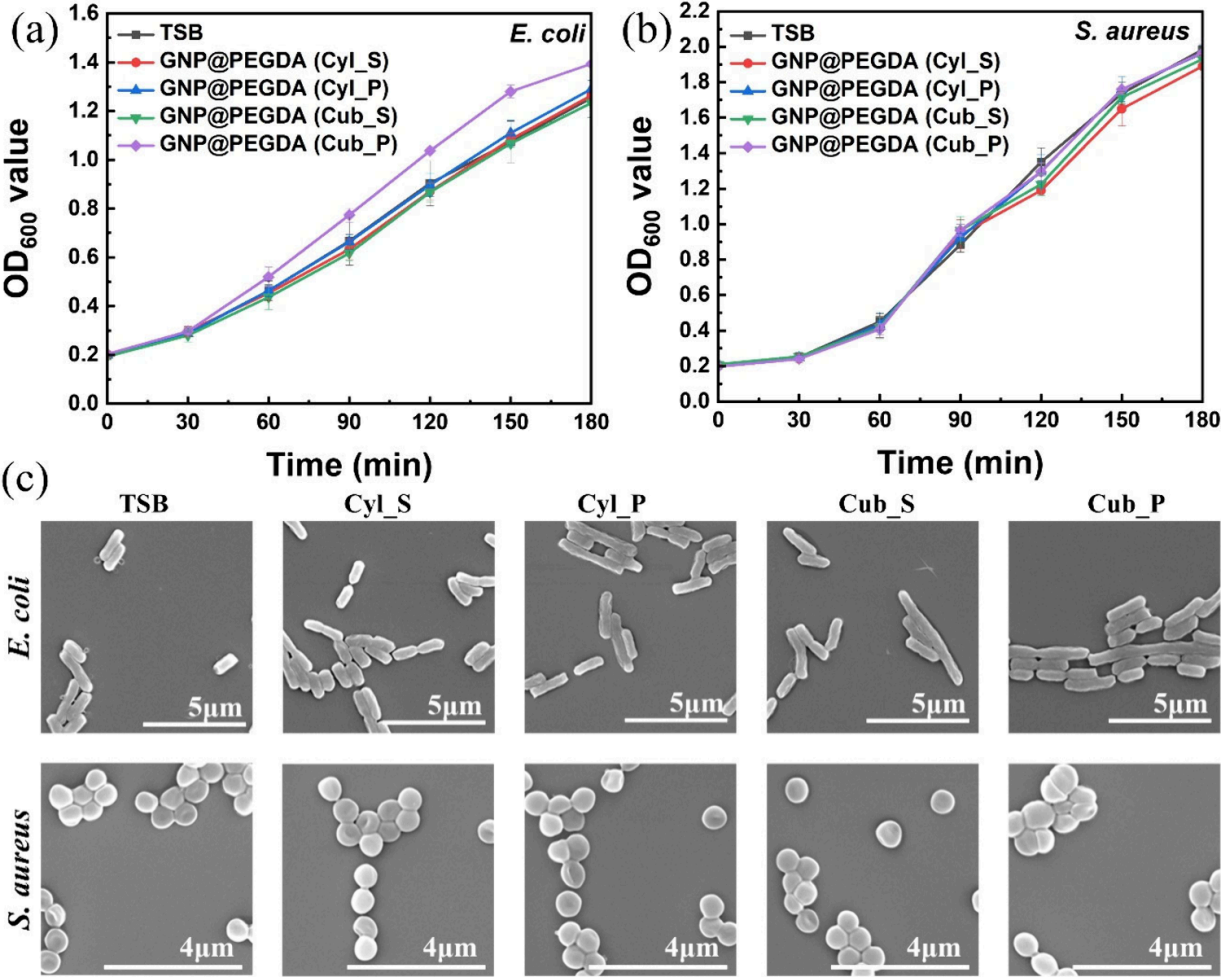
Antibacterial activity without irradiation. OD regrowth curve of (a) *Escherichia coli* (*E. coli*) and (b) *Staphylococcus aureus* (*S. aureus*) incubated with different shapes of GNP@PEGDA scaffolds without irradiation. (c) SEM images of unirradiated bacteria. The TSB group consisted of untreated bacteria.

### 3.5 Antibacterial Activity of GNP@PEGDA with NIR Radiation

To further verify the antibacterial activity of GNP@PEGDA scaffolds against the model bacteria of *E. coli* and *S. aureus* with irradiation, a growth curve was calculated. According to Figure 5, both *E. coli* and *S. aureus* in TSB as control groups began to grow from 30 min initially and rose sharply through the next 3 h, which demonstrated proclivity toward growth. After 3 h, OD values reached the highest values of 1.3 for *E. coli* and 2.0 for *S. aureus* (Figure 5a,b). When the bacteria were treated with GNP@PEGDA scaffolds under NIR light, the growth curve obviously remained unchanged at 0.2 until 2.5 h for all shapes of scaffold and then increased to 0.3 in the last 30 min. There was no significant difference in the OD between the two kinds of bacteria (*E. coli* and *S. aureus*) within 3 h. With NIR light absorption, GNP@PEGDA of different shapes efficiently produced heat and reached 63–70 °C after 10 min of treatment. This exceeded the temperature limit for *E. coli* and *S. aureus* to grow, which is only in the range 35–40 °C. This produced a tremendous effect on the bacteria with no gradual bacterial growth during the 3 h incubation period in contrast to the situation without irradiation. Therefore, the growth of bacteria was entirely inhibited. Furthermore, an SEM analysis was performed to confirm the antibacterial effect of GNP@PEGDA scaffolds. Untreated bacterial cells were smooth, rod-shaped for *E. coli* and were sphere-shaped for *S. aureus*, as well as having a uniform size and distribution (Figure 5c). In contrast, bacterial cell walls were morphologically damaged after NIR laser induction with the combination of the bacterial solution and all GNP@PEGDA shapes. The cell morphology of *E. coli* treated with the solid cuboid and porous cylinder shapes was severely damaged followed by the solid cylinder and porous cuboid; *S. aureus* was also badly harmed in terms of morphology with all four shapes of the solid cuboid, porous cuboid, solid cylinder, and porous cylinder. All bacteria showed disruption of the bacterial membrane and cellular integrity. The results revealed that the solid form had greater antibacterial activity and photothermal activity which destroyed cell walls and cell membranes after NIR treatment.

**Figure 5:**
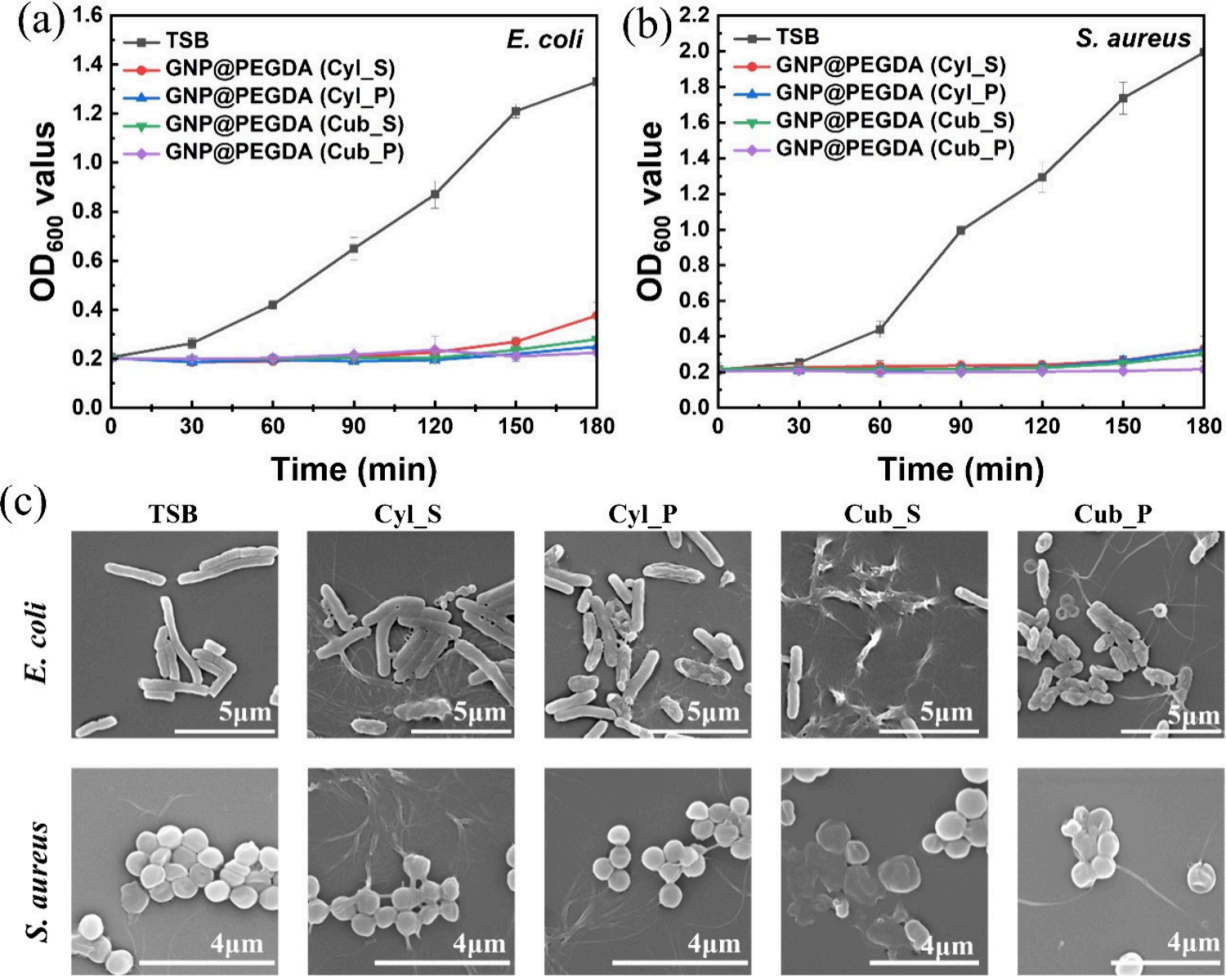
Antibacterial activity. Growth curves of (a) *Escherichia coli* (*E. coli*) and (b) *Staphylococcus aureus* (*S. aureus*) incubated with GNP@PEGDA scaffolds after 10 min of irradiation. (c) SEM images of bacterial treatment.

### 3.6 Agar Plate analysis

To confirm the antibacterial effect of GNP@PEGDA scaffolds combined with NIR light, an agar plate assay was conducted. Plate Count Agar (PCA) is a nonselective medium used to count the quantity of live bacteria. As shown by the agar plate assay in Figure 6, this was used to represent the situation in real conditions from bacterial growth curves. On the agar plate (Figure 6a), TSB was the control group without irradiation of the GNP@PEGDA scaffold and shows that there was bacterial growth of *E. coli* and *S. aureus* after being subjected to radiation light for 10 min. This was supported by the survival rate trends (Figure 6b, c) which showed that 100% of the bacteria were still alive in the control group for both types of bacteria. In comparison, GNP@PEGDA scaffolds in shapes of a solid cylinder, porous cylinder, solid cuboid, and porous cuboid produced exceptional results, with no bacterial growth on the agar plate after 24 h of incubation. This reveals that GNP@PEGDA scaffolds of different shapes with irradiation treatment can significantly kill both *E. coli* or *S. aureus* bacteria compared to agar plates without light absorption. This was confirmed by the survival rate trends (Figure 6b,c), as no colonies were seen on the agar plates after treatment with different scaffold shapes. This indicates that the GNP@PEGDA scaffolds possess higher antibacterial activities with laser light absorption for both bacterial strains. To sum up, the GNP@PEGDA scaffolds demonstrated remarkable photothermal antibacterial properties due to the synergistic effects of GNPs and PEGDA matrix. Upon exposure to NIR laser irradiation, GNPs efficiently convert light into localized heat, generating a photothermal effect that disrupts bacterial structures. SEM images reveal significant morphological damage to bacterial cell walls caused by this photothermal mechanism. The combination of bacterial solution and all GNP@PEGDA scaffold shapes under NIR laser treatment leads to irreversible membrane damage, suggesting effective bacterial eradication at the cellular level. Furthermore, agar plate analysis confirms the scaffold’s photothermal antibacterial activity. Under NIR laser irradiation, no bacterial growth was observed on the agar plates, demonstrating the photothermal inhibition of bacterial proliferation.

**Figure 6:**
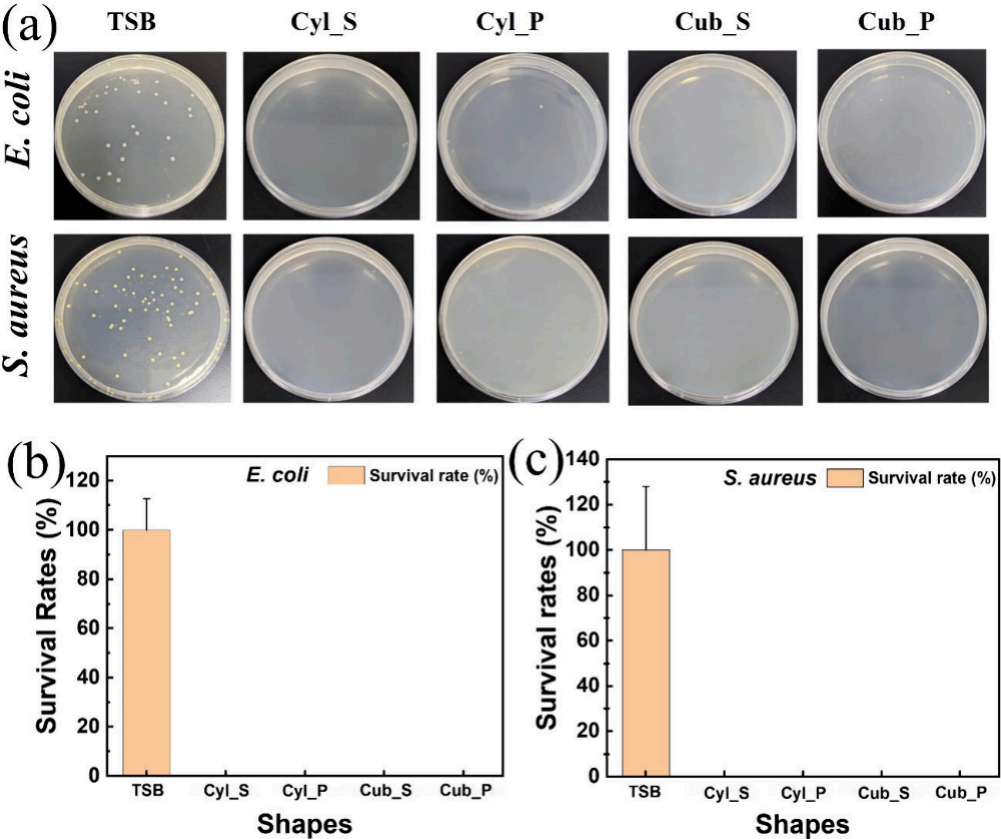
Appearance of plate counting assay: bacterial colonies of *Escherichia coli* (*E. coli*) and *Staphylococcus aureus* (*S. aureus*) with different shapes of the GNP@PEGDA scaffold after irradiation and incubation for 24 h (a) followed by enumeration of living bacteria of *E. coli* (b) and *S. aureus* (c).

## 4 Conclusions

In this study, we present a groundbreaking advancement in healthcare hygiene by introducing the GNP@PEGDA composite material, a product of blending graphene nanoplatelets (GNPs) with poly­(ethylene glycol) diacrylate (PEGDA) through 3D printing. This innovative material exhibited an exceptional photothermal performance, especially in porous structures, effectively addressing the limitations of traditional disinfection methods. The combination of surface antibacterial and photothermal technologies proved to be highly effective in inhibiting bacterial proliferation without relying on chemical disinfectants, thereby reducing environmental burdens. The research findings, validated through microbial cultures, temperature elevation curves, photothermal activity assessments, SEM observations, and agar plate assays, underscore the composite material’s promising antibacterial efficacy. The integration of 3D printing technology enhanced the efficiency of these technologies, providing a novel and efficient approach to improving hygiene in healthcare materials and facilities. This study not only highlights the significance of 3D printing but also underscores the potential of photothermal technology, offering a compelling direction for the future of healthcare surface disinfection methodologies.
